# Clinical and cardiac magnetic resonance findings in post-COVID patients referred for suspected myocarditis

**DOI:** 10.1007/s00392-021-01929-5

**Published:** 2021-08-26

**Authors:** Philipp Breitbart, Alexander Koch, Marco Schmidt, Annett Magedanz, Edelgard Lindhoff-Last, Thomas Voigtländer, Axel Schmermund, Rajendra H. Mehta, Holger Eggebrecht

**Affiliations:** 1 Cardioangiological Center Bethanien (CCB), Im Prüfling 23, 60389 Frankfurt, Germany; 2 Division of Cardiology and Angiology II, University Heart Center Freiburg-Bad Krozingen, University Hospital Freiburg, Bad Krozingen, Germany; 3 AGAPLESION Bethanien Hospital, Frankfurt, Germany; 4 Duke Clinical Research Institute (DCRI), Durham, NC USA

**Keywords:** COVID-19, Myocarditis, Myocardial inflammation, Cardiac imaging, MR-tomography

## Abstract

**Objectives:**

We assessed possible myocardial involvement in previously cardiac healthy post-COVID patients referred for persisting symptoms with suspected myocarditis.

**Background:**

Prior studies suggested myocardial inflammation in patients with coronavirus-induced disease 2019 (COVID-19). However, the prevalence of cardiac involvement among COVID patients varied between 1.4 and 78%.

**Methods:**

A total of 56 post-COVID patients without previous heart diseases were included consecutively into this study. All patients had positive antibody titers against SARS-CoV-2. Patients were referred for persistent symptoms such as chest pain/discomfort, shortness of breath, or intolerance to activity. All patients underwent standardized cardiac assessment including electrocardiogram (ECG), cardiac biomarkers, echocardiography, and cardiac magnetic resonance (CMR).

**Results:**

56 Patients (46 ± 12 years, 54% females) presented 71 ± 66 days after their COVID-19 disease. In most patients, the course of COVID-19 was mild, with hospital treatment being necessary in five (9%). At presentation, patients most often reported persistent fatigue (75%), chest pain (71%), and shortness of breath (66%). Acute myocarditis was confirmed by T1/T2-weighed CMR and elevated NTpro-BNP levels in a single patient (2%). Left ventricular ejection fraction was 56% in this patient. Additional eight patients (14%) showed suspicious CMR findings, including myocardial edema without fibrosis (*n* = 3), or non-ischemic myocardial injury suggesting previous inflammation (*n* = 5). However, myocarditis could ultimately not be confirmed according to 2018 Lake Louise criteria; ECG, echo and lab findings were inconspicuous in all eight patients.

**Conclusions:**

Among 56 post-COVID patients with persistent thoracic complaints final diagnosis of myocarditis could be confirmed in a single patient using CMR.

**Graphic abstract:**

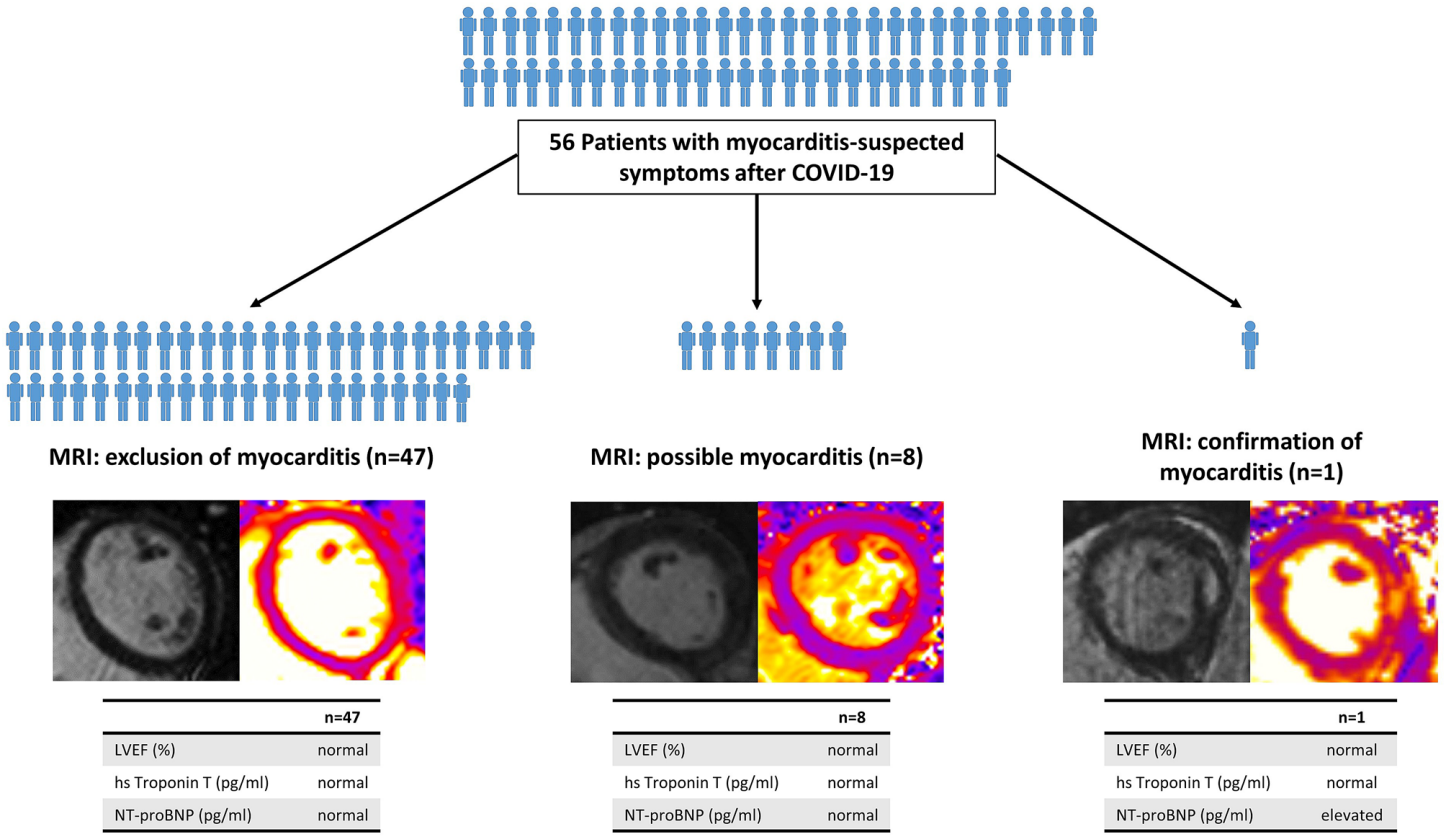

## Introduction

Early in the severe acute respiratory syndrome coronavirus-2 (SARS-CoV-2) pandemic, individual case reports and smaller case series suggested that coronavirus-induced disease 2019 (COVID-19) can lead to deterioration of cardiac function in patients with previous cardiovascular diseases [1–3].

Previous studies using cardiovascular magnetic resonance (CMR) imaging which is considered the gold standard for non-invasive myocarditis diagnosis [4], provided conflicting results on the prevalence of COVID-19 associated myocarditis ranging between 1.4% [5] and as high as 58–78% of patients [6, 7].

In clinical practice we are often confronted with post-COVID patients without previous cardiac diseases who suffer from persistent thoracic complaints, exertional dyspnea and/or exercise intolerance, even months after their SARS-CoV-2 infection. Data specifically for this increasing group of patients are scarce. Therefore, we aimed to investigate the prevalence of myocardial inflammation in post-COVID patients without previous cardiac diseases, referred for suspicion of myocarditis.

## Methods

### Study population

Consecutive patients with no history of previous cardiac disease who were referred with persistent symptoms suspicious of myocarditis such as chest pain/discomfort, shortness of breath, intolerance to activity, dizziness and/or palpitations since their COVID-19 disease were included into the study. Diagnosis of COVID-19 was based on a previous positive polymerase chain reaction (PCR) test and positive SARS-CoV-2 IgM/IgG antibodies directed against the viral nucleocapsid antigen (Elecsys® Anti-SARS-CoV-2, Roche; negative = cut-off index (COI) < 1). Exclusion criteria were (1) known or newly diagnosed non-inflammatory heart disease, (2) contraindications for performing CMR with contrast media (e.g., claustrophobia, contrast medium allergy, glomerular filtration rate ≤ 30 ml/min/1.73 m^2^, implants or devices without admission for CMR) or insufficient image quality, (3) hemodynamic instability or (4) pregnancy/lactation.

All patients gave their written informed consent for the anonymized use of clinical, procedural, and follow-up data. The study was approved by the local institutional review board (ethics committee of the Landesärztekammer Hessen) and complies with the Declaration of Helsinki. The study was sponsored by the Foundation of the Working Group of Leading Cardiological Hospital Doctors e.V. (ALKK).

### Diagnostic procedures

All patients completed a dedicated questionnaire to assess their current complaints as well as the symptoms during the acute COVID-19 disease, including a 10-point visual analog scale for individual quantification of COVID-19 severity from “0” (no symptoms) to “10” (most severe course). Patients underwent a standardized cardiologic assessment including 12-lead electrocardiogram (ECG), echocardiographic evaluation of systolic and diastolic left ventricular as well as valvular function, and assessment of laboratory cardiac markers including high-sensitivity troponin T (hsTnT), creatine kinase (CK), and N-terminal pro-b-type natriuretic peptide (NT-proBNP).

### Cardiac magnetic resonance imaging

All patients underwent the CMR examination on a 1,5 Tesla scanner (Magnetom Aera©, Siemens Healthcare GmbH, Erlangen, Germany). Every scan was performed and evaluated jointly by a cardiologist and a radiologist, each certified as having the maximum level of qualification in cardiac MRI from their societies (German Cardiac Society, German Radiological Society). In a standardized protocol, the localizers were followed by evaluation of left ventricular function parameters using a semi-automatic approach (syngo.via ©, Siemens Healthcare GmbH, Erlangen, Germany). Using cine images in short-axis (SAX) stack, 2-/3- and 4-chamberview, we evaluated the left ventricular ejection fraction (LVEF) as well as left ventricular end-diastolic volume index (LVEDVI), left ventricular end systolic volume index (LVESVI), left ventricular stroke volume index (LVSVI) and myocardial mass (MM). In addition, T2 darkblood TIRM-Sequences were performed in three views (axial from base to apex, 4- and 2-chamber-view).

T1 mapping was performed using electrocardiographically triggered modified look-locker inversion-recovery (MOLLI) sequence with a 5(3)3 acquisition (five heartbeats of acquisition are followed by three recovery heartbeats which are followed by another three acquisition heartbeats) on three short-axis (basal, midventricular, apical). T2 maps were acquired in the same planes as the T1 maps using the SIEMENS MyoMaps product sequence (Siemens Healthcare GmbH, Erlangen, Germany), which is T2-prepared. T2 map is calculated from three single-shot acquisitions at different times of T2 preparation (0 ms, 25 ms, 55 ms). Furthermore, T1 maps were created with a 4(1)3(1)2 acquisition on three short-axis (basal, midventricular, apical) approximately 15 min after contrast agent application to create extracellular volume (ECV) map and T1 error map. Generally, we performed a semi-automatic quantification (cvi42©, Circle Cardiovascular Imaging Inc., Calgary, Canada) of T1, T2 and ECV maps for every of the 16 myocardial segments.

Late gadolinium enhancement (LGE) (0.2 mmol/kg Gadolinium [Dotarem©, Guerbet GmbH, Sulzbach, Germany]) imaging was acquired in the same planes as cine imaging with a phase-sensitive inversion-recovery sequence.

### CMR imaging analysis

Increased T1 values were defined according to internal reference values as greater than 1077 ms (basal), 1083 ms (midventricular) or 1081 ms (apical) and T2 mapping values greater than 50 ms (basal), 51 ms (midventricular) or 53 ms (apical), respectively. These sequence-specific cutoffs have previously been defined as laying 2 SDs above the respective means in a healthy population. Significant abnormalities were defined as greater than 1145 ms (basal), 1153 ms (midventricular) or 1146 ms (apical) for T1 and greater than 54 ms (basal), 56 ms (midventricular) or 59 ms (apical) for T2, using 4 SDs (based on earlier international publications (7)) above those means.

According to the 2018 Lake Louis criteria, myocarditis is diagnosed if both of the main criteria are positive: proof of (1) myocardial edema (T2 mapping or T2 darkblood TIRM-Sequences) and (2) non-ischemic myocardial injury (abnormal T1, ECV or LGE). Only one fulfilled criterion still supports the diagnosis of acute myocardial inflammation in an appropriate clinical scenario. These patients are classified as “MRI possible myocarditis”.

### Statistical analysis

We performed all statistical analyses with SPSS software, Version 25.0 (IBM Corp., Armonk, NY, USA). Categorical data are described as frequencies or percentages and continuous variables as mean with standard deviation or median with interquartile range. We defined a *P* value < 0.05 as statistically significant in all tests.

## Results

Between November 2020 and March 2021, 62 post-COVID patients with no history of previously heart disease were referred for evaluation of persistens symptoms suspicious of myocarditis. In five of them we had to interrupt the CMR-scan due to progressive claustrophobia symptoms and the image quality was poor in 1. 56 patients were included (mean age 46 ± 12 years, 54% female) for final analysis. Baseline characteristics are presented in Table 1.

**Table 1:** Baseline characteristics of the entire study population

		All patients (*n* = 56)
Age	(years)	45.7 ± 12.2
Female	*n*	30 (53.6)
Any previous medication	*n*	24 (42.9)
Beta blocker	*n*	4 (7.1)
AT1RB	*n*	4 (7.1)
ACEI	*n*	5 (8.9)
Statin	*n*	4 (7.1)
Acute COVID-19 symptoms		
Fever	*n*	34 (60.7)
Cough	*n*	33 (58.9)
Limb pain	*n*	38 (67.9)
Sore throat	*n*	20 (35.7)
Cold	*n*	20 (35.7)
Headache	*n*	44 (78.6)
Shortness of breath	*n*	43 (76.8)
Loss of smell of taste	*n*	41 (73.2)
Pneumonia	*n*	4 (7.1)
Pulmonary artery embolism	*n*	1 (1.8)
Hospital treatment due to COVID-19	*n*	5 (8.9)
Acute COVID-19 severity score	(0–10)*	4.7 ± 2.2
Time between positive swab and MRI	(days)	70.7 ± 65.9
Current symptoms		
Fatigue	*n*	42 (75.0)
Shortness of breath	*n*	37 (66.1)
Chest pain	*n*	40 (71.4)
Palpitations	*n*	4 (7.1)
Headache	*n*	3 (5.4)
Insomnia	*n*	3 (5.4)
Cough	*n*	3 (5.4)
SARS-CoV2-antibody level	COI	61.6 ± 56.7

Values are mean ± standard deviation or *n* (%)

*AT1RB *Angiotensin-II subtype I receptor blocker, *ACEI *angiotensin converting enzyme inhibitor, *COI *cut-off index, *COVID-19 *coronavirus-induced disease 2019, *SARS-CoV-2 *severe acute respiratory syndrome coronavirus-2

*Severity level 0 means no symptoms, level 10 means maximum symptoms

The most common acute COVID-19 symptoms were headache (79%), shortness of breath (77%), and loss of smell or taste (73%). A hospital treatment due to COVID-19 was necessary in five patients (9%), but no patient received mechanical ventilation.

Patients presented 71 ± 66 days after their COVID-19 disease. All patients had positive SARS-CoV2-antibody (61.6 ± 56.7 COI). Most often reported complaints included persisting fatigue (75%), chest pain (71%), and shortness of breath (66%).

Electrocardiogram, echocardiographic, and laboratory chemistry findings are summarized in Table 2. Three patients (5%) showed ST-changes suspicious of myocarditis. On echocardiography, all patients had a normal ejection fraction (mean 67 ± 7%) without evidence of relevant diastolic dysfunction. In a single patient, mild pericardial effusion was noted. The mean cardiac blood values were 4.3 ± 1.9 pg/ml for hsTnT, 94.9 ± 53.3 U/l for CK, and 34.5 [23.0;68.5] pg/ml for NT-proBNP.

**Table 2:** Electrocardiogram, echocardiography, and cardiac markers of the entire study population

		All patients (*n* = 56)
ECG findings		
Heart rate	Beats / min	69.1 ± 11.8
QRS time	Ms	92.6 ± 8.6
Myocarditis suspicious ST-changes	*N*	3 (5.4)
Bradycardia	*N*	4 (7.1)
Tachycardia	*N*	1 (1.8)
Echocardiographic findings		
Ejection fraction	(%)	67.2 ± 6.5
Left ventricular end-diastolic diameter	Mm	46.8 ± 4.6
E/E'		5.6 ± 1.5
Wall movement disorders	*n*	2 (3.6)
Pericardial effusion	*n*	1 (1.8)
High-sensitivity troponin T	pg/ml	4.3 ± 1.9
Creatine kinase	U/l	94.9 ± 53.3
NT-ProBNP	pg/ml	34.5 [23.0;68.5]

Values are mean ± standard deviation, median [interquartile range] or *n* (%)

*ECG *electrocardiogram, *NT-proBNP *N-terminal pro-b-type natriuretic peptide

*Severity level 0 means no symptoms, level 10 means maximum symptoms

### CMR findings

CMR results are presented in Table 3. Using the 16 myocardial segment model, 13 of all 928 segments (1%) could not be evaluated in T1 maps due to artifacts or thin myocardium, 44 of 928 segments (5%) in T2 maps. Mean T1 value was 1016.0 ± 28.2 ms, mean T2 value 46.9 ± 3.8 ms. Overall, a pathological T1 relaxation time was found in four of the evaluable segments (0.4%) and a pathological T2 relaxation time in 16 segments (2%). Late gadolinium enhancement was detectable in seven patients (12%) with subepicardial (five patients), subendocardial (one patient), and intramyocardial (one patient) localisation.

**Table 3:** Magnetic resonance imaging findings of the entire study population

		All patients (*n* = 56)
Left ventricular end-diastolic volume index (LVEDVI)	ml/m^2^	76.4 ± 13.8
Left ventricular end systolic volume index (LVESVI)	ml/m^2^	29.1 ± 7.4
Left ventricular stroke volume index (LVSVI)	ml/m^2^	47.3 ± 8.3
Myocardial mass (MM)	g/cm	0.7 ± 0.2
Ejection fraction	%	62.3 ± 5.0
Suspicious TIRM-findings	*n*	2 (3.6)
T1 values		
Mean	ms	1016.0 ± 28.2
Basal	ms	1011.9 ± 28.7
Mid-ventricular	ms	1012.7 ± 29.2
Apical	ms	1021.7 ± 35.6
T2 values		
Mean	ms	46.9 ± 3.8
Basal	ms	46.4 ± 2.5
Mid-ventricular	ms	47.2 ± 2.7
Apical	ms	48.8 ± 3.1
Extracellular volume (ECV) map	%	27.5 ± 3.4
Late gadolinium enhancement (LGE)	*n*	7 (12.5)
Subepicardial	*n*	5 (8.9)
Subendocardial	*n*	1 (1.8)
Intramyocardial	*n*	1 (1.8)

Values are mean ± standard deviation or *n* (%)

**Fig. 1:**
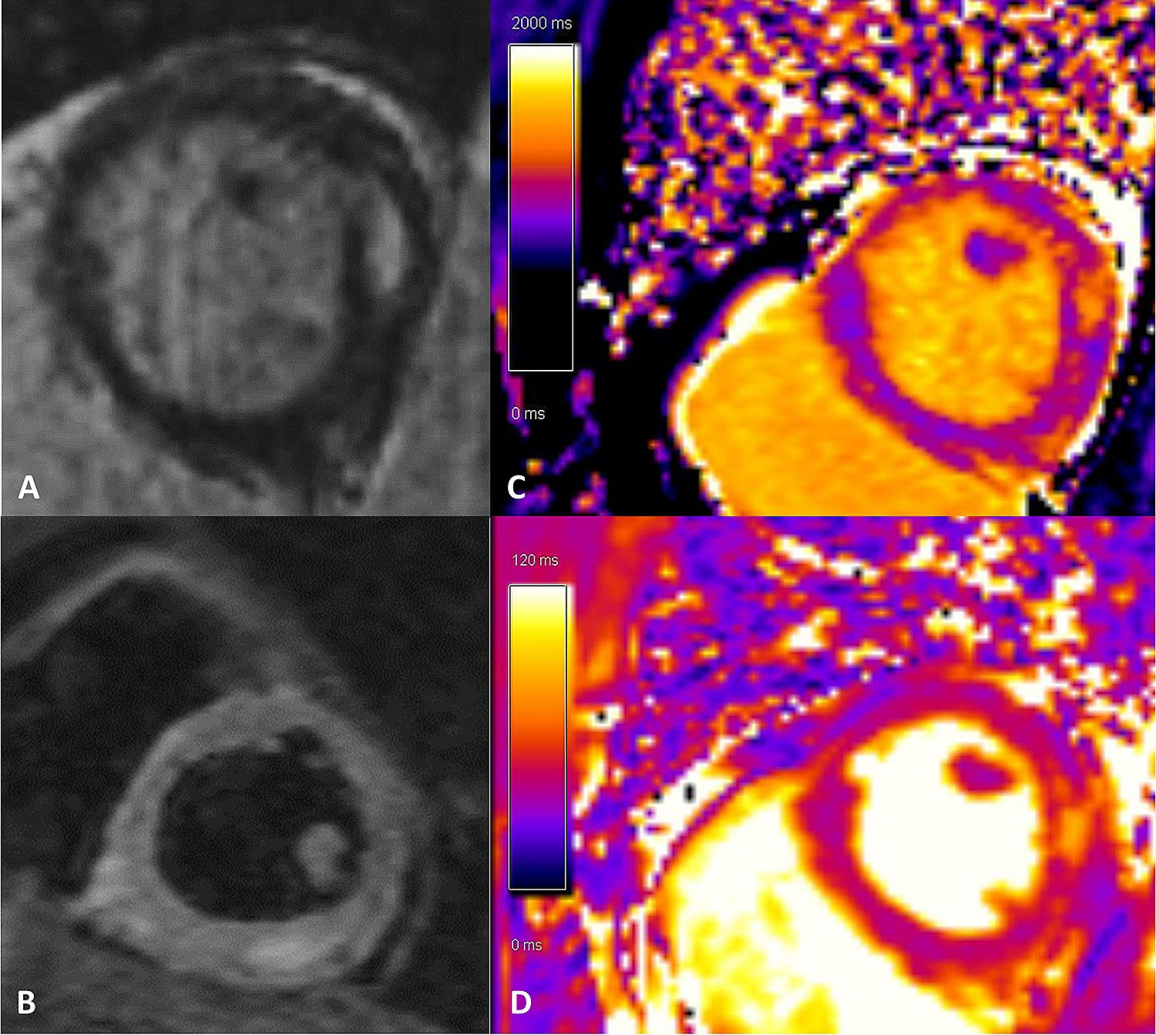
MRI diagnosis of an acute myocarditis. MRI-findings of the patient who fulfilled both Lake Louise criteria with abnormal findings with emphasis basal inferolateral: non-ischemic myocardial fibrosis proven by intramyocardial late gadolinium enhancement **(A)** and increased T1 times **(C)** as well as signs of a myocardial edema with pathological TIRM-Sequence **(B)** and increased T2 relaxation time **(D)**

The results of the various cardiac examinations of all patients with abnormal MRI-findings are summarized in Table 4. One patient (2%) fulfilled both Lake Louise criteria with intramyocardial LGE as well as increased T1 and T2 times (Fig. 1). In this patient, NT-proBNP was increased to 967 pg/ml, but ejection fraction was within the normal range (56%).

**Table 4:** Electrocardiogram, echocardiographic, laboratory chemistry findings, and MRI-finding of patients, who fulfilled at least one Lake Louise criteria

		Both Lake Louise criteria fulfilled	One Lake Louise criteria fulfilled (*n* = 8)							
		(*n* = 1)	Patient A	Patient B	Patient C	Patient D	Patient E	Patient F	Patient G	Patient H
Myocarditis suspicious ST-changes	*n*	None	None	None	None	None	None	None	Yes	None
*Echocardiographic findings*										
Ejection fraction	(%)	64	74	66	65	68	64	69	77	58
Left ventricular end-diastolic										
Diameter	mm	45	50	42	40	46	44	49	49	58
E/E'		8	6	5	10	5	5	8	5	7
Wall movement disorders	*n*	None	None	None	None	None	None	None	Yes	None
Pericardial effusion	*n*	None	None	None	None	None	None	None	Yes	None
*Laboratory chemistry findings*										
High-sensitivity troponin T	pg/ml	7	5	3	4	4	3	7	5	14
Creatine kinase	U/l	149	100	90	69	48	29	63	86	49
NT-ProBNP	pg/ml	967	93	73	69	88	26	32	22	65
Antibody level	COI	25.9	45	175	24	1.3	1.4	33	10	64
MRI-findings										
Ejection fraction	(%)	56	73	66	64	67	55	67	67	59
Edema in TIRM-Sequence		Yes	None	None	None	None	None	Yes	None	None
T1 values										
Mean	ms	1128	972	995	999	1003	1034	1013	1066	1015
Basal	ms	1118	981	999	993	1011	1040	1030	1037	1009
Mid-ventricular	ms	1124	974	980	998	990	1027	1011	1072	985
Apical	ms	1161	959	1006	1010	1005	1006	993	1105	1059
T2 values										
Mean	ms	59	44	48	46	50	52	47	45	46
Basal	ms	59	43	50	45	49	48	46	46	46
Mid-ventricular	ms	60	44	50	47	49	51	47	45	46
Apical	ms	61	44	50	47	51	55	49	44	46
Extracellular volume (ECV) map	%	35	26	27	30	30	28	28	27	26
Late gadolinium enhancement (LGE)		Intramyocardial	Subepicardial	None	Subepicardial	Subepicardial	None	None	Subepicardial	Subepicardial

NT-proBNP N-terminal pro-b-type natriuretic peptide

*COI *cut-off index

In eight (14%) patients, only one of both criteria was fulfilled: three patients (5%) showed myocardial edema and five (9%) non-ischemic myocardial injury (Table 4; Fig. 2). None of the three patients with myocardial edema (two patients with increased T2 relaxation times, one with pathological TIRM-Sequence) had conspicuous echocardiographic, electrocardiogram or laboratory chemistry findings.

The five patients with non-ischemic myocardial injury showed subepicardial LGE localisations and a mean EF of (mean EF 68%. Three of these patients had normal findings in the further examinations. One patient showed wall movement disorders, pericardial effusion and myocarditis suspicious ST-changes, but the cardiac blood values were normal. Three patients had borderline hsTnT value without any echocardiographic or electrocardiogram abnormalities. All five findings were consistent with healed inflammation.

## Discussion

Cardiac involvement in COVID-19 has been reported previously with conflicting data on prevalence and clinical relevance. Our study investigated post-COVID patients without previous heart disease presenting with persistent symptoms suspicious of myocarditis to a large cardiology practice. Using a comprehensive diagnostic approach including CMR, final diagnosis of myocarditis was infrequent and confirmed in a single out of 56 patients (2%).

Previous studies have assessed myocardial involvement based on CMR findings in COVID-19 patients. The prevalence of abnormal CMR findings varied widely, between 1.4% and 78% [5–9]. The broad range may be explained by differences in diagnostic assessment as well as patient selection (e.g., competitive student athletes with an asymptomatic course versus patients with previous cardiac diseases requiring mechanical ventilation). Importantly, only the minority of the published studies used the 2018 Lake Louise criteria when interpreting the CMR findings [5, 8]. Increased T1 values or LGE both may not be specific for acute myocardial inflammation, but may also reflect previous myocardial injury of all ages [10]. The potential interference of previous cardiac disease is important to note, since up to 56% of hospitalized COVID-19 patients had pre-existing cardiovascular disease [11]. A recent CMR evaluation revealed comparable LGE rates in athletes with and without COVID-19 history [12]. A pathological study examining 40 hearts from patients dying of COVID-19 found myocyte necrosis in 35%, which were mostly caused by microthrombi in epicardial coronary artery, myocardial capillaries, arterioles, and small muscular arteries [13].

On the other hand, myocardial oedema detected by T2 darkblood TIRM-Sequences or T2 mapping is not specific for myocarditis or inflammation in general [10].Therefore, the interpretation of all abnormal CMR findings as cardiac COVID-19 involvement may lead to an obvious overestimation of the diagnosis of myocarditis.

We used strict criteria, requiring fulfillment of both the 2018 Lake Louise criteria as well as pathological cardiac markers to diagnose myocarditis. Our findings are in line with autopsy reports as well as a recent CMR analysis in athletes recovering from COVID-19, suggesting that acute myocarditis is rare [5, 14]. In the single patient with confirmed myocarditis but also in the additional eight requiring only one criterion of the modified Lake Louise criteria we found normal LV ejection fraction, which is an important prognostic marker in myocarditis [15]. However, the long-term significance of the abnormal CMR finding after COVID-19 recovery is not yet known.

In the eight patients of our study fulfilling only one of both myocarditis criteria, myocarditis cannot be ruled out completely. Particularly, myocardial edema which was observed in three of them could be related to increased vascular permeability mediated by endothelial angiotensin converting enzyme 2 (ACE2) which is an established functional receptor by which SARS-CoV-2 enters host target cells [16–18]. Of note, hsTNT as well as NTpro-BNP were normal in all of them.

The remaining five patients showed subepicardial LGE localisation as a marker of non-ischemic myocardial injury, which may be unspecific as we do not have CMR assessment before COVID-19. The echocardiographic and laboratory chemistry findings were inconspicuous, so we postulated healed inflammation that had passed at an unclear point in time.

### Clinical competencies

A high number of patients recovered from SARS-CoV-2 infections present with persistent thoracic complaints suspicious of myocarditis. Our results suggest that definitive myocarditis is uncommon in post-COVID patients without previous cardiac disease. A single patient fulfilled all criteria of the modified Lake Louise criteria for diagnosing myocarditis. In additional eight patients myocarditis could neither be confirmed nor excluded. However, relevant myocarditis appeared unlikely as LV function was not impaired and laboratory markers of cardiac injury were inconspicuous. These findings are important to soften fears of long-term cardiac sequelae with possible psychosocial consequences in younger patients. In addition, the routine use of expensive (CMR) imaging in the diagnostic assessment of post-COVID patients with chest complaints may only be rarely needed. Our results suggest that a primary strategy using more conventional modalities such as ECG, echocardiography, and cardiac biomarkers may be sufficient to identify patients who could benefit from CMR.

## Limitations

Since we excluded patients with known or newly diagnosed non-inflammatory heart disease, our findings are not transferrable to this group. However, acute SARS-CoV-2 infections affect a large number of younger patients with no prior illness, to which our findings should apply. Our study is limited by the restricted sample size and the use of a 1,5 Tesla scanner for CMR. Nevertheless, the robustness of the finding that myocardial inflammation is rare in our cohort is supported by the coherent laboratory results as well as the echocardiographic and CMR findings.

## Conclusion

Definitive myocarditis was detected in a single out of 56 post-COVID patients without previous cardiac diseases presenting with persistent thoracic complaints suspicious of myocarditis. These data may provide some reassurance to symptomatic patients post-COVID regarding their risk of myocardial inflammation and argues against routine use of expensive modalities as CMR in all these patients.

## Data Availability

Not applicable.

## Abbreviations

CK: Creatine kinase
CMR: Cardiovascular magnetic resonance
COI: Cut-off index (COI)
COVID-19: Coronavirus-induced disease 2019
ECG: Electrocardiogram
ECV: Extracellular volume
hsTnT: High-sensitivity troponin T
LGE: Late gadolinium enhancement
NT-proBNP: N-terminal pro-b-type natriuretic peptide
PCR: Polymerase chain reaction
SARS-CoV-2: Severe acute respiratory syndrome coronavirus-2
